# Coregulators determine androgen receptor activity in prostate cancer

**DOI:** 10.1042/BSR20253197

**Published:** 2025-08-20

**Authors:** Kerim Yavuz, Nathan A. Lack

**Affiliations:** 1 Vancouver Prostate Centre, Department of Urologic Sciences, University of British Columbia, Vancouver, BC V6H 3Z6, Canada; 2 Department of Medical Pharmacology, School of Medicine, Koç University, İstanbul 34450, Turkey; 3 Koç University Research Centre for Translational Medicine (KUTTAM), Koç University, İstanbul 34450, Turkey

**Keywords:** androgen receptor, coregulator proteins, epigenetics, gene expression and regulation, prostate cancer

## Abstract

The androgen receptor (AR) is the main driver of nearly all prostate cancer (PCa). It alters gene expression by binding to specific cis-regulatory elements on the DNA. Where the AR binds in the genome determines what genes are expressed. However, the AR cistrome is not static and dramatically changes during PCa initiation and progression to activate distinct transcriptional programs that fuel disease growth and therapeutic resistance. Emerging evidence suggests that these changes in DNA binding are not caused by chromatin accessibility but rather from interactions with AR coregulators. These proteins influence AR at every step of its activity and play a critical role in DNA binding and gene activation. These context-specific coregulator interactions can stabilize AR binding with DNA that has low- to moderate-affinity and also affect locus-specific epigenetic modifications to promote transcription. Given their critical role in this process, alterations to coregulator proteins define the normal and oncogenic cistrome and profoundly affect AR-mediated gene transcription. In this review, we aim to provide a new perspective on the role of AR coregulators in transcriptional activity, how these interactions evolve through different stages of PCa and their potential as therapeutic targets in advanced disease.

## Introduction

The growth and proliferation of almost all prostate cancer (PCa) is driven by the androgen receptor (AR) (1,2). Given its critical function, targeting AR signaling is the main treatment for metastatic or recurrent PCa (3). However, while most tumors are initially responsive, resistance eventually develops in nearly all patients as the cancer advances to castration-resistant prostate cancer (CRPC). This aggressive form of the disease has a median survival of only ~2 years following diagnosis (4). While a subset of these resistant tumors differentiates into an AR-negative neuroendocrine state (~15–20%), the vast majority of CRPC tumors still remain dependent on AR signaling even when the cancer has been treated with potent AR pathway inhibitors (ARPIs) including enzalutamide, abiraterone, or apalutamide (5–11). Yet while AR is essential at all stages of PCa, its activity dramatically changes during disease progression (12–14). During PCa initiation, the AR binds to DNA sites that are proposed to induce cell proliferation (15,16). In CRPC, the AR-binding sites (ARBS) are even further expanded to reactivate a latent prostate developmental program (14) that strongly correlates with clinical outcome (17). Notably, the massive change in the AR cistrome—the genome-wide universe of ARBS—is highly recurrent across AR-expressing tumors, suggesting that this is a near-universal feature. Understanding how AR activity changes during PCa progression is critical to develop more effective treatments.

AR regulates gene transcription in response to androgen stimulation (18). When bound by androgens in the cytoplasm, the AR dissociates from chaperone heat shock proteins and translocates into the nucleus (11). In the nucleus, the AR binds to DNA at ARBS located predominantly in intronic and intergenic regions. These distal ARBS primarily regulate the expression of AR target genes through enhancer activity (19). Enhancers are cis-regulatory elements that affect gene expression through physical interactions with target gene promoters that occur by chromatin looping. Chromatin loops are DNA structures that are formed through the actions of architectural proteins like cohesin complex and bring the distal regulatory sites in close proximity to facilitate functional interactions through activator proteins such as the Mediator complex (20,21). These interactions increase transcription through both the recruitment of activator proteins and stabilization of RNA polymerase II (RNA Pol II) at target gene promoters (22). Given the essential role of these genomic elements in mediating expression across all tissues and organs, there is strict regulation for the proper functioning of enhancers. There is clear clinical evidence that enhancer dysregulation contributes to the development of PCa due to its roles in cell proliferation and regulation of the expression of a number of tumor suppressor and proto-oncogenes (23,24). Due to the significant role of AR in PCa, characterization of AR-driven enhancers and how they act on downstream genes can identify potential therapeutic targets.

While ARBS function as enhancers to regulate gene expression, their activity is largely defined by coregulator proteins that modulate DNA binding by the AR and transcriptional outcomes. Coregulators are proteins with context-specific actions that can help to stabilize AR binding to DNA, affect local chromatin environments to induce transcription, or contribute to RNA Pol II activation. Because of their critical role in these processes, alterations to coregulator proteins can have a substantial impact on gene regulation (25–27). Understanding the role of these coregulators on AR-mediated transcription is essential to identify novel therapeutic targets and/or diagnostic markers. In this review, we summarize the role of AR coregulators in gene transcription and describe how these proteins contribute to the initiation and progression of PCa.

## AR activation

AR is a ligand-inducible transcription factor that comprises three main domains: a ligand-binding domain (LBD), which is found on the C-terminus of the protein and binds to androgens; a DNA-binding domain (DBD) that recognizes binding motifs on the DNA; and an N-terminal domain (NTD) that is responsible for the transactivation through interactions with coregulator proteins and basal transcription machinery (28). The LBD and DBD are separated by the Hinge domain which provides flexibility to the protein and contains the nuclear localization signal (NLS) (29).

**Figure 1:**
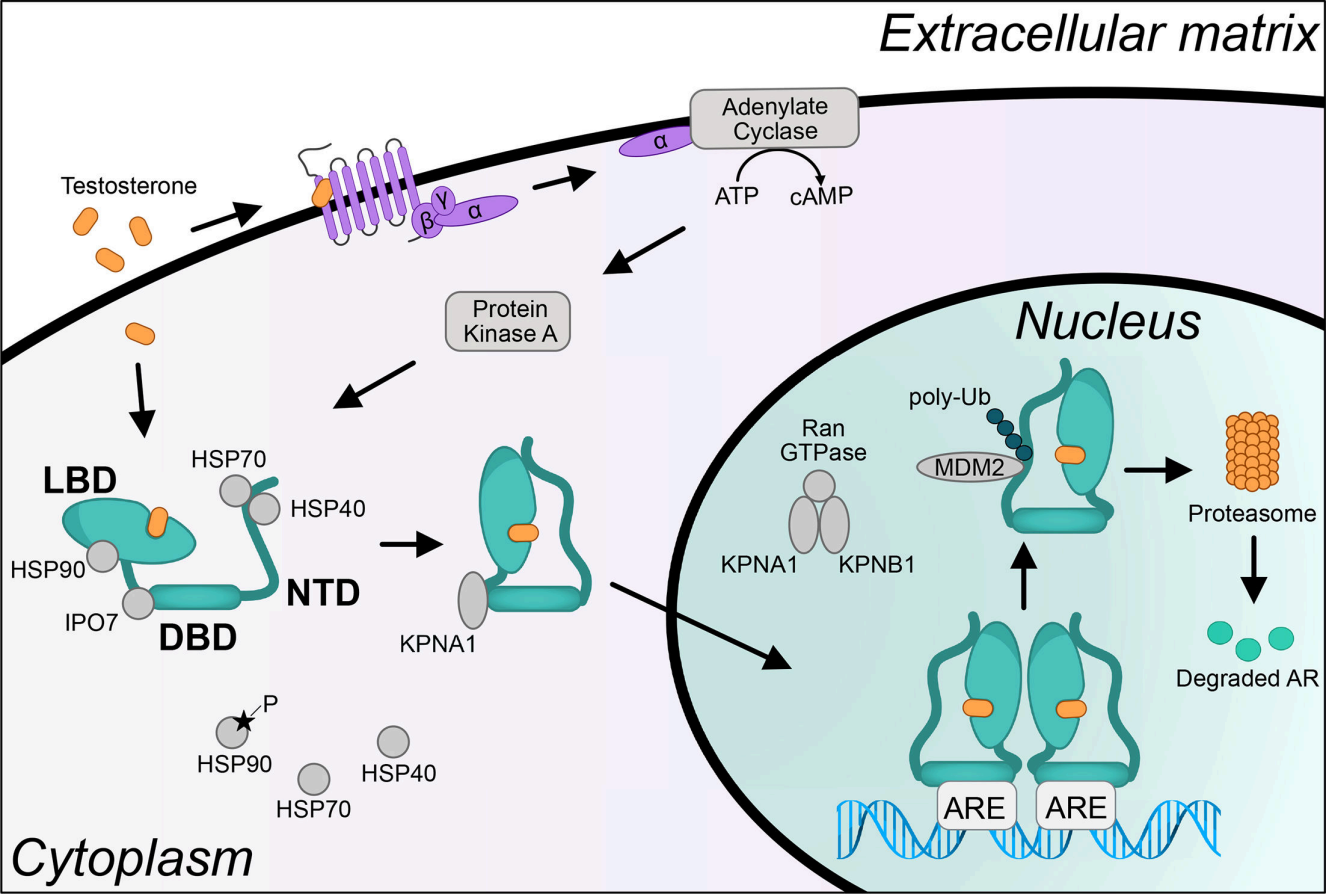
Schematic representation of AR activation. Inactive AR is found in the cytoplasm bound to chaperone proteins that keep it in a permissive conformation to allow ligand binding. When androgens bind to the LBD, it induces a conformational change that results in N/C interaction. Simultaneously, androgens bind to a GPCR on the membrane that increases cAMP levels in the cell and activates protein kinase A (PKA). PKA phosphorylates HSP90, causing its dissociation from AR. Upon ligand binding, IPO7, which masks the NLS dissociates from the AR and AR is translocated into the nucleus by KPNA1. In the nucleus, KPNA1 forms a complex with KPNB1 and Ran GTPase, releasing AR in the nucleus. AR homodimerizes and binds to DNA on its cognate response element, regulating the expression of its target genes. The turnover of AR is mediated by the ubiquitinating activity of MDM2 E3-ubiquitin ligase, directing it to proteasomal degradation. AR, androgen receptor; ARE, androgen response element; cAMP, cyclic adenosine monophosphate; DBD, DNA-binding domain; IPO7, importin 7; LBD, ligand-binding domain; NTD, N-terminal domain.

AR becomes activated upon binding to androgenic ligands such as testosterone or the more potent dihydrotestosterone (Figure 1). In the unbound state, AR is found in the cytoplasm and is associated with molecular chaperones such as Hsp70, Hsp40, and Hsp90 which keep the apo-protein in a state permissive to ligand binding (30). When androgens bind, the AR undergoes a conformational change that induces an N–C interaction between the NTD and LBD through the N-terminal amino acids FQNLF (F: Phenylalanine, Q: Glutamine, N: Asparagine, and L: Leucine) (31). In the absence of the ligand, this N–C interaction is inhibited by chaperone proteins (32). Simultaneously with AR activation, androgens also bind to a G-protein-coupled receptor (GPCR) that causes increased levels of cyclic adenosine monophosphate which activates Protein Kinase A. This then phosphorylates Hsp90 causing its dissociation from AR (33,34). Upon dissociation of chaperone proteins, AR translocates to the nucleus where it dimerizes and binds to DNA to activate transcription. AR homodimerization has been shown to occur through both the DBD and LBD (35). While controversial, recent studies have suggested that AR monomers and dimers may activate opposing biological processes. At low testosterone concentrations, the monomers are proposed to activate the mTOR pathway, while higher concentrations cause AR to dimerize and induce expression of canonical AR target genes (36). Nuclear translocation of AR is regulated by the importin family proteins including importin 7 that dissociates from AR upon ligand binding, causing the NLS to be exposed (37). The AR NLS is initially recognized by importin α (KPNA1), which translocates the protein to the nucleus where the complex interacts with importin β (KPNB1). This causes binding of Ran GTPase, which dissociates the importins and leaves the AR in the nucleus (38). Once in the nucleus, AR binds to specific DNA regions containing its cognate response element. From both protein-based Systematic Evolution of Ligands by Exponential Enrichment (SELEX) studies and chromatin immunoprecipitation followed by sequencing (ChIP-seq) assays, AR binding is found to enrich at specific DNA motifs including a 15-nt canonical androgen response element (ARE) that consists of the inverted repeat of a hexameric binding site of 5′-AGAACA-3′ separated by a spacer of three random nucleotides (19,39). Each half of the palindromic ARE is recognized by a single AR monomer through one of the two zinc finger domains in the DBD, while the remaining zinc finger domain of each monomer interacts with the other forming an AR homodimer on DNA (40). This sequence can also be recognized by other nuclear receptors including glucocorticoid receptor (GR) (41). However, as identified through sequence analyses and electrophoretic mobility shift assays of the enhancers of AR target genes such as rat probasin, sex-limited protein, or KLK3, an AR-binding motif composed of a direct repeat, instead of the inverted repeat, 5′-AGAACAnnnAGAACA-3′, is only recognized by AR and therefore is called the selective ARE (41–43). The second zinc finger in the DBD confers this AR-specific binding, as replacement of this zinc finger with the second zinc finger of GR causes loss of AR binding to selective AREs (44). AR also binds at some half AREs (hARE) that contain only a single hexameric sequence (45–47). Whether AR binds to these half-sites as a monomer or a dimer is a topic of debate. It has been shown *in vitro* that AR monomers associate with hAREs (48). Although DNA-footprinting assays suggest hAREs might be bound by a single AR molecule (49), ChIP-seq assays in cells expressing AR only in monomeric form are necessary to elucidate the mode of AR binding on hAREs. While the motifs of AR and other transcription factors correlate with DNA binding, the presence of the motif is not enough by itself to infer binding, as the majority of accessible regions with these motifs are not bound (50). Simply put, most accessible sites that contain an ARE are not bound by AR (51). This suggests an additional layer of regulation that determines which sites AR binds to on DNA.

Once bound to enhancers on DNA, AR exerts its transcriptional action through interactions with various coregulator proteins. These enhancers interact with target gene promoters through pre-established chromatin loops that are stabilized upon AR activation (52). These stabilized AR-bound loops up-regulate a variety of genes, notably those involved in prostate gland secretion and lineage-specific functions (53). Similar to other nuclear receptors, ligand binding induces a conformational change that forms a binding site that can selectively interact with coregulator proteins. While the interaction between coregulators and other nuclear receptors occurs through short helical peptides containing LXXLL (L: Leucine, X: any amino acid) (54,55), AR preferentially interacts with peptides containing an FXXLF (F: Phenylalanine, L: Leucine, X: any amino acid). This protein motif is also found in the AR NTD and was shown to be important in both coregulator and N/C interactions (56,57).

## Transcriptional coregulators of AR

Nuclear hormone receptors exert their genomic functions through interactions with transcriptional coregulators that affect numerous regulatory functions (58–60). These can alter transcriptional activation and are broadly classified by their ability to increase (co-activators) or decrease (co-repressors) gene expression. These definitions, however, are far too simplistic as many coregulators both increase and decrease gene expression in a locus-specific mechanism (61). Dysregulation of AR activity by transcriptional coregulators can activate oncogenic pathways resulting in the initiation and progression of PCa. While AR interacts with hundreds of proteins, these coregulators are broadly thought to have three main roles in AR transcriptional activity: Determining the cistrome profile, altering the local chromatin environment and activating RNA Pol II to increase gene expression.

### AR coregulators that affect DNA binding

**Figure 2:**
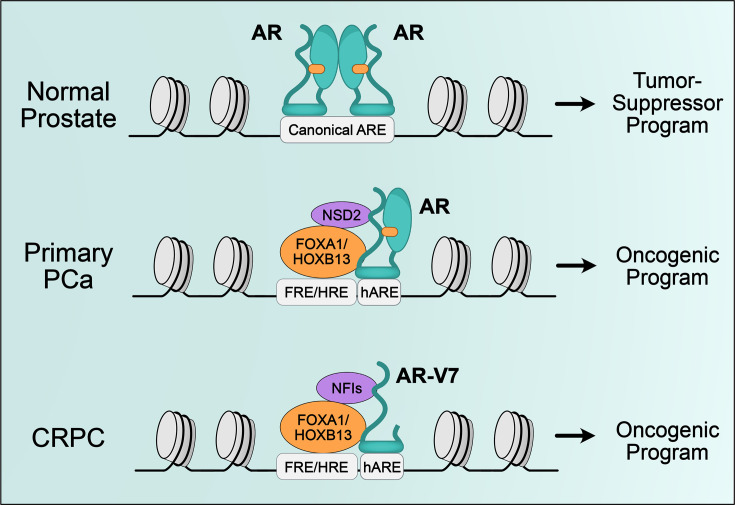
AR cistrome alterations by pioneer factors. Majority of the ARBS in the normal prostate tissue contain canonical AREs, while in primary PCa, the coregulators with pioneering functions such as FOXA1 and HOXB13 alter the AR cistrome to enrich occupancy of composite AR:Coregulator sites to drive oncogenic programs. These sites contain a half ARE (hARE), potentially bound by an AR monomer, and coregulator motifs such as forkhead response element (FRE) or homeobox response element (HRE). NSD2 also reprograms AR cistrome to enrich occupancy of AR:FOXA1 and AR:HOXB13 sites, contributing to tumorigenesis. In CRPC, NFI family proteins increase AR-V7 binding to AR:FOXA1 and AR:HOXB13 sites contributing to enzalutamide resistance. AR, androgen receptor; ARE, androgen response element; CRPC, castration-resistant prostate cancer; PCa, prostate cancer.

Where AR binds in the genome determines which genes are expressed. Given such a critical function, it is unsurprising that AR’s interaction with DNA is strictly regulated. However, this process is highly complex. While specific sequences or motifs correlate with AR binding, there is increasing experimental evidence that coregulators strongly influence the AR cistrome (Figure 2). One important class of coregulators that play a critical role in AR-mediated transcription is pioneer factors, which can bind to their target sequences even if they are compacted by histones (62). While the classification of coregulators has been distinct from pioneer factors as they are recruited to chromatin through protein–protein interactions, recent reports suggest that these proteins are far more complex and can also have pioneer activity that can increase chromatin accessibility (62–65). Further complicating these classical definitions, pioneer factors, such as FOXA1, HOXB13, and GATA2, have been shown to recruit AR to distinct ARBS and stabilize their binding at specific sites (66,67). Given this pleiotropic activity that is highly context- and locus-specific, in this review, we chose to describe both the transcriptional co-activators and the well-known pioneer factors as coregulators.

#### FOXA1

FOXA1 is a DNA-binding forkhead protein that plays an important role as an AR coregulator. It has been demonstrated to act as a pioneer factor for nuclear hormone receptors including AR and estrogen receptor (ER) (68), facilitating chromatin accessibility by binding to compacted nucleosomes and displacing the linker H1 histone (69). The recent cryo-EM structure of FOXA1–DNA revealed that FOXA1 can bind to the edge of the nucleosome, bending the linker DNA away from the linker histone, thereby promoting the unpacking of neighboring nucleosome array (70). In an ER+ breast cancer cell line, FOXA1 was shown to promote ER binding to DNA, while its knockdown caused loss of euchromatin (71). Disruption of FOXA1’s pioneering activity was shown to affect breast cancer tumor development (72). For AR, FOXA1 was shown to physically interact with DBD/Hinge domains and similar to its relationship with ER, it has been proposed to regulate the transcription of canonical AR target genes such as prostate-specific antigen (*KLK3*) through its pioneering activity (66). Importantly, FOXA1 reprograms the AR cistrome in PCa and its overexpression, either alone or in combination with HOXB13, can push the AR towards a more tumor-like cistrome (15). However, FOXA1 may contribute more than just pioneer factor activity to AR binding. Knockdown of this protein causes a massive expansion of ARBS, suggesting a role for FOXA1 to recruit AR at specific sites (67,73,74). Explaining this dual role, an equilibrium-dependent mechanism was proposed (67). When AR levels exceed those of FOXA1, AR binds to sites containing canonical AREs. However, when FOXA1 levels increase to those similar to AR, it drives the AR cistrome to the sites that contain both FOXA1 and AR motifs. Finally, when FOXA1 levels drastically exceed those of AR, it then causes an increase in chromatin accessibility that allows AR to gain low-affinity binding sites. Mutations to FOXA1 can also affect the AR cistrome (75) and are enriched in primary PCa, contributing to increased AR transcriptional activity and promoting proliferative and pro-tumorigenesis pathways. Further mutations in the C-terminal domain (CTD) and structural variants in the *FOXA1* gene locus are observed in CRPC and activate the WNT-signaling pathway important in metastasis. Alterations of the AR cistrome by FOXA1 have clinical implications. Those ARBS with canonical AREs are proposed to have tumor suppressing activity in healthy prostate tissue while composite motifs containing both AR:FOXA1 motifs are enriched in primary PCa ARBS (41,76). These AR-coregulator interactions that change DNA binding profiles can be exploited as a therapeutic target. In a recent study, the AR coregulator NSD2 was shown to reprogram the AR cistrome to enrich occupancy of chimeric AR:FOXA1 or AR:HOXB13 half-sites in PCa (77). The use of an NSD2 PROTAC degrader that disrupted the AR–FOXA1 interaction caused rapid PCa cell death (77). This suggests that identification of AR coregulators that have a role in cistrome reprogramming can potentially offer novel therapeutic targets.

#### HOXB13

HOXB13 is a homeobox coregulator of AR that is primarily expressed only in the prostate tissue (78) and, similar to other posterior HOX members, is involved in prostate development (79). Germline HOXB13 variants have been shown to be strong risk factors for PCa (80–82). HOXB13 physically interacts with the AR DBD to drive the expression of its target genes (83). Its coregulatory activity is bidirectional, facilitating both the up- and down-regulation of AR target genes by modulating DNA binding (83). HOXB13 is suggested to be a pioneer factor that preferentially binds methylated CpG dinucleotides (84). In line with this, recent work revealed that HOXB13 interacts with SMARCD2, a member of the SWI/SNF nucleosome remodeling complex, to increase chromatin accessibility of HOXB13 binding sites that contain AR and FOXA1 motifs (85). Similar to FOXA1, overexpression of HOXB13 in an non-neoplastic prostate cell line pushed the AR cistrome to a more tumor-like profile (15). While this suggests a role of HOXB13 in the initiation of PCa, it has also been shown to be important in progression to CRPC. Interactions with HOXB13 and AR-V7, a constitutively active variant of AR that emerges in CRPC, up-regulate genes associated with cancer progression and metastasis (86). HOXB13 also increases androgen-independent growth of LNCaP cells through the activation of E2F signaling pathway (87). HOXB13 germline variants were also shown to have an AR-independent role in PCa progression, through lipid accumulation that promotes cell motility and tumor metastasis (88). These clear dependencies to HOXB13 in both androgen-dependent and -independent PCa suggest that it could be a potential therapeutic drug target.

#### GATA2

GATA2 is a pioneer factor that is important to the transcriptional activity of AR. It helps recruit AR to enhancers and is proposed to also stabilize chromatin loops from enhancers to the target genes (89,90). GATA2 has been shown to not only regulate the expression of wildtype AR and constitutively active AR variants but also colocalize with AR and FOXA1 on chromatin to recruit transcriptional co-activators such as p160-family proteins and CBP/p300 (91). Studies investigating the hierarchical relationship between FOXA1 and GATA2 in the androgen signaling axis revealed the interplay between these two factors in AR gene regulation. While both coregulators co-occupy a large portion of genome-wide ARBS, more regions are co-occupied by FOXA1 than GATA2 (89,90). AR knockdown had no effect on FOXA1 or GATA2 occupancy, indicating that the binding of these coregulators precedes AR binding (89). However, AR binding to DNA does stabilize the binding of these pioneering factors (89). The majority of the GATA2-occupied ARBS are also bound by FOXA1 with less than 10% of ARBS bound by GATA2 alone. This is proposed to occur as FOXA1 itself initiates the reprogramming of the GATA2 cistrome toward a forkhead motif containing ARBS (90). The combinatorial activity of these two coregulators is implicated in PCa drug resistance through the expression of a multidrug-resistance-associated protein (ABCC4) (89,92–94). In addition, GATA2 was shown to play a role in darolutamide resistance in PCa through its pioneering activity (95). GATA2 increases AR’s association with chromatin, and through rewiring of the AR cistrome, leads to an overexpression of metastasis-related genes such as *ALCAM* (95), demonstrating its clinical relevance in the progression of PCa.

#### NFI family proteins

Nuclear factor I (NFI) family proteins are AR coregulators that aid in AR-mediated transcription by co-binding to ARBS together with AR and FOXA1 (96). The NFI protein family, consisting of NFIA, NFIB, NFIC, and NFIX, binds to DNA at a consensus sequence of 5′-TTGGCnnnnnGCCAA-3′ (97). NFI family proteins are suggested to repress the expression of AR target genes, and knockdown of NFIB is shown to cause benign prostate hyperplasia, suggesting a role in the initiation of PCa (98). All NFI family proteins are expressed in LNCaP, an androgen-dependent PCa cell line, at various levels, and they interact with both FOXA1 and AR, suggesting that they aid in the oncogenic cistrome reprogramming (96). In addition to the proposed role in PCa initiation, the FOXA1–NFIs–AR complex has been suggested to contribute to PCa progression as well, as the NFI motif is highly enriched at those sites that gain accessibility upon enzalutamide resistance (99). These sites were shown to be bound by the constitutively active AR-V7, and the knockdown of NFIB and NFIX resulted in significantly diminished transcriptional activity (99). Even though the mechanism of action of NFI family proteins is yet to be elucidated, the potential pioneer function (100,101) suggests that they might stabilize AR-V7 binding to DNA during late-stage PCa or, as proposed earlier, could interact with AR–FOXA1 complex to stabilize the protein–DNA interaction on chromatin (99). Overall, these findings suggest an important role of FOXA1–NFIX signaling in the initiation and progression of PCa, and it offers a potential therapeutic target for the treatment of advanced PCa.

All of the above-mentioned pioneer factors alter chromatin accessibility. However, their expression also dramatically affects the AR cistrome to activate oncogenic programs and drive PCa progression. While the overexpression of FOXA1 and HOXB13 pushes AR cistrome to a more tumor-like profile, pioneering activity of GATA2 allows AR to bind to enhancers of metastasis and treatment resistance associated genes, thereby contributing to PCa progression. Similar to GATA2, NFI family proteins, specifically NFIB and NFIX, also take part in the late-stage PCa, acting to contribute to AR-V7 binding to DNA upon enzalutamide resistance. However, it is noteworthy that FOXA1 appears to be the major factor, both determining AR cistrome by itself and interacting with other pioneer factors to rewire their cistromes and help them recruit AR. Overall, this suggests that in addition to potential pioneering activity, these proteins also stabilize AR binding to DNA.

### AR coregulators in transactivation

**Figure 3:**
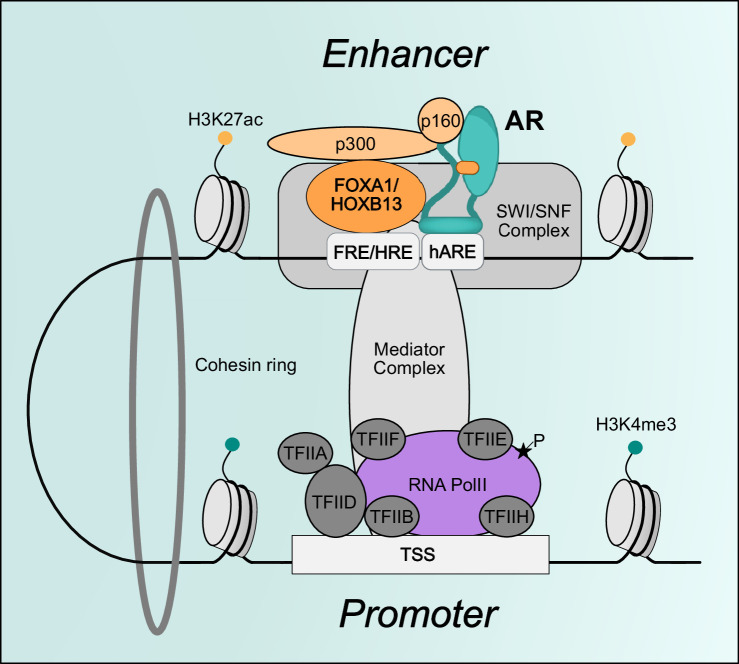
AR-mediated transcription. AR interacts with various transcriptional coregulators to alter the chromatin environment to a transcriptionally permissive state and facilitate RNA Pol II recruitment and activation. AR interacts with p160 family proteins at distal enhancers, which recruit CBP/p300 to deposit H3K27ac enhancer-associated histone modification. Nucleosome remodeling complexes such as SWI/SNF complex recognizes acetylated histones and interacts with AR for nucleosome remodelling and increased chromatin accessibility at ARBS. The AR-bound enhancer and AR-target gene promoters are bridged together by Mediator complex through a chromatin loop formed by the Cohesin complex. Mediator complex helps to initiate transcription by stabilizing PIC and phosphorylating RNA Pol II at Ser5 position on the 7-amino acid repeat found in the CTD. AR, androgen receptor; FRE, forkhead response element; HRE, homeobox response element; RNA Pol II, RNA polymerase II; TSS, transcription start site.

AR coregulators that play a crucial role in the transcriptional activation can be divided into two categories: those that prepare the chromatin environment to confer a transcriptionally permissive state, and those that recruit and activate RNA Pol II (Figure 3). Coregulators in the first category are mainly epigenetic modifiers that modify the chromatin to enable pre-initiation complex (PIC) to bind and recruit RNA Pol II. These proteins function in writing, reading, and/or erasing epigenetic modifications, increasing the chromatin accessibility and altering the methylation state of the DNA. The coregulators that aid in the recruitment of RNA Pol II and its initial activation are mainly parts of large protein complexes that connect enhancers and promoters. The actions of these two groups of proteins result in a direct or indirect impact on the expression of AR target genes, and in this section, we will describe the functions of AR coregulators that take part in AR-mediated transactivation.

#### AR coregulators in epigenetic modifications

Spatiotemporal regulation of gene expression requires switching of chromatin between transcriptionally permissive and repressive states. One of the main mechanisms through which this is achieved is histone modifications, which can change DNA compaction around the histones and recruit chromatin remodeling factors for repositioning of nucleosomes (102). AR interacts with various histone modifying enzymes to activate the transcription of its target genes, and the histone modifications have been shown to have critical functions at AR-driven enhancers (103). Among the AR coregulators with histone modifying activity are the p160 family proteins, also known as the steroid receptor co-activator (SRC) protein family. This family is composed of three proteins—SRC-1, -2, and -3—that interact with the AR-NTD to activate target gene expression (104–106). p160 family proteins also interact with the LBD of AR, but this interaction was shown to activate transcription to a weaker extent (107). These proteins activate transcription by direct or indirect histone acetylation, which increases the negative charge on the histones and causes DNA to unwind from the histone, making it accessible for additional transcription factors to bind (102). SRC-3 was shown to have direct histone acetyltransferase (HAT) activity and also stabilized the interaction between AR and p300 (27,108). Through these actions, SRC family proteins significantly contribute to AR activity, and the dysregulation of their function has been shown to have a role in the progression of PCa. The overexpression of both SRC-1 and SRC-2 contributes to the ligand-independent activity of AR and drives the progression to CRPC (109,110).

p300 is a HAT that interacts with the AR on the NTD (27). It is recruited to chromatin and enables AR-mediated transcription (111). p300 is implicated in CRPC due to its role in maintenance of androgen-independent AR activity. AR interacts with p300 through its phosphorylated Serine 81 residue, whose phosphorylation is catalyzed through the activity of the cyclin-dependent kinase 9 subunit of the positive transcription elongation factor (112–114). This facilitates recruitment of p300 to AR-mediated enhancers such as *KLK2 *or* KLK3* enhancers to mediate histone acetylation to form a transcriptionally active chromatin state (115). It was shown that in CRPC, CDK1 expression is increased, which leads to increased S81 phosphorylation of AR, resulting in sustained AR activity through p300 recruitment in low androgen levels (115,116). p300 also provides an open chromatin environment for the binding of FOXA1 in PCa, and its inhibition causes loss of chromatin accessibility of sites co-occupied by AR and FOXA1 (117). Due to its role in AR signaling, as well as DNA damage repair pathways (118), p300 has been shown to be a potential therapeutic target for the treatment of CRPC (119,120).

Histone methylation is also shown to contribute to gene regulation and is associated with both repression and activation (102,121). Removal of repressive marks, including H3K9me3 and H3K27me3, is critical for gene expression, and various lysine-specific demethylases were shown to be important for AR transcriptional activity. For example, KDM1A, KDM3A, and KDM4B were identified as AR coregulators, acting through demethylation of repressive histone marks in AR target promoters (122–126). These, along with other histone demethylases KDM4A, KDM5A, and KDM3B, were shown to contribute to the progression to CRPC by promoting androgen-independent PCa growth (123,127). Arginine methylation of histones has also been shown to contribute to gene regulation. Co-activator arginine methyltransferase 1 indirectly interacts with AR on the *KLK3* promoter and contributes to increased gene expression (128). Overall, the regulation of AR target genes is strongly influenced by alterations of chromatin state through histone modifying AR coregulators.

In addition to post-translational modifications (PTMs) of histones, there is extensive evidence that PTMs of both AR (129–131) and coregulators such as SRC-3 and MED1 (132,133) can contribute to the transcriptional activities of these key proteins important in PCa initiation and progression.

#### AR coregulators in chromatin accessibility

Chromatin accessibility is critical for transcription factors to bind to their cognate response elements and is a highly dynamic feature that plays an important role in the establishment and maintenance of cellular identity (134–136). It is achieved by the activity of nucleosome remodeling complexes that bind to the DNA and evict or slide nucleosomes to make regions accessible for transcription factors to bind (137). While ARBS accessibility is necessary for gene expression and affected by AR coregulators’ recruitment, the majority of ARBS are accessible even before androgens activate the AR (49,52,138). However, during the transition between primary to advanced PCa, new ARBS gain accessibility and remain open, independent of hormone stimulation (139). AR has been shown to interact with various nucleosome remodeling complexes in PCa, suggesting a role for these in AR-driven transcription. For example, AR-mediated transcription of *KLK3* was shown to be dependent on the ATPase activity of hBRM subunit of SWI/SNF ATP-dependent chromatin remodeling complex (140). AR interacts with the SWI/SNF complex through the catalytic subunit SMARCA4 and increases accessibility of a subset of ARBS upon androgen induction, suggesting that it is recruited by AR to increase accessibility of the target enhancers (141). The accessibility of ARBS was also shown to be affected by indirect coregulators of AR. For example, CHD1, a chromatin modifying protein, was shown to have a role in increasing accessibility of ARBS even though it does not directly interact with AR (142). CHD1 was shown to colocalize with pioneer factors such as FOXA1 and HOXB13 on AR enhancers, suggesting that these proteins might have a role in its recruitment (142). These findings suggest a role of AR coregulators in increasing chromatin accessibility to activate AR-mediated transcription.

The functional cooperation between AR and chromatin modifying enzymes can affect ARPI resistance in PCa. It was reported that BRD2/4 and ATAD2, both co-activators of AR, mediate increased chromatin accessibility and AR association with chromatin in CRPC compared with benign prostate hyperplasia, potentially contributing to enzalutamide resistance (143). In addition to the recruitment of BRD2/4 and ATAD2 to ARBS with gained accessibility, it was shown that AR overexpression also causes up-regulation of these factors in CRPC, forming a positive feedback loop that results in AR cistrome expansion (143). It was also shown that ARBS that are co-occupied by BRD4 and SMARCA4 can also gain GR binding upon enzalutamide treatment and maintain signaling of AR target genes (144). Overall, these findings suggest that despite ARBS being accessible before ligand activation, AR interacts with chromatin remodeling proteins to further modulate binding site accessibility driving CRPC progression.

#### AR coregulators in DNA methylation

DNA methylation is a key regulator of gene expression and is associated with transcriptional repression. Therefore, demethylation of AR-target promoters is an important step in transcriptional activation (145). This primarily occurs through an AR-independent mechanism as there are limited interactions between AR and DNA methyltransferase enzymes or demethylases. However, there are examples of AR interacting proteins affecting DNA methylation. The DNA demethylase TET2 was shown to interact with AR to regulate the expression of tumor-suppressor genes, suggesting that it has a role in tumor suppression and its loss can lead to the progression of PCa (146). However, this mechanism remains to be elucidated, and further studies are needed to identify those factors that contribute to ARBS demethylation.

#### AR coregulators in RNA polymerase II recruitment and activation

In addition to providing the local environment needed to form the PIC, AR coregulators also contribute to the activation of RNA Pol II. Transcriptional initiation at promoters occurs through a series of events that forms a platform to enable RNA Pol II recruitment, binding, and activation. Briefly, on a TATA-box containing promoter, the PIC is formed through TFIID, which recognizes the TATA-box located at −31 to −26 bp relative to the transcription start site, as well as the Initiator (Inr) sequence located at −2 to +4 bp. In a TATA-less promoter, the initial recognition takes place on the Inr and downstream promoter element, located at +28 to +33 bp. TFIID is stabilized on DNA by the binding of TFIIA and TFIIB to form the platform upon which RNA Pol II binds. TFIIF recruits RNA Pol II and, together with TFIIE, positions the nontemplate strand so that the initial transcription bubble forms. TFIIH acts as helicase to unwind the DNA strand and phosphorylates RNA Pol II, and TFIIH-associated kinase Cdk7 phosphorylates RNA Pol II at the Ser5 position of the 7-amino acid repeat in the CTD (147).

Various protein complexes have been shown to interact with AR to induce the formation of PIC and the recruitment of RNA Pol II to its target gene promoters. Among these, the Mediator complex has a critical role in RNA Pol II recruitment. Mediator is a large protein complex that is composed of 26 subunits and functions to bridge enhancers and promoters by conveying signals from transcription factors to RNA Pol II (148). It also aids in the initiation of transcription through recruitment of PIC to target gene promoters (149). The AR–Mediator complex interaction is critical for AR-driven transcription. Interactions between MED1 and AR induce the recruitment of the Mediator complex to the promoter of *KLK3* and enable expression (150). It was proposed that Mediator facilitates RNA Pol II recruitment to the KLK3 promoter by tracking it along a chromatin loop extending from an upstream enhancer (151). According to this model, RNA Pol II is initially deposited on the *KLK3* enhancer through p160 and p300 complex proteins and then tracks through the chromatin loop formed by the Mediator complex between the *KLK3* enhancer and promoter. This then recruits the PIC to the promoter to initiate the transcription of *KLK3* in an androgen-inducible manner (151). This model is supported by an additional study which elucidated the role of p160 complex proteins in transferring RNA Pol II from *KLK3* enhancer to promoter (152). In this study, SRC-3, an AR coregulator, was shown to be necessary for the recruitment of RNA Pol II to the *KLK3* enhancer. Once bound, RNA Pol II CTD is phosphorylated, facilitating RNA Pol II transfer from the *KLK3* enhancer to promoter (152). These suggest an alternative mechanism by the Mediator complex, through which RNA Pol II is recruited to AR target gene promoters after initially binding to AR-driven enhancers. In addition, AR–Mediator complex interaction was shown to induce formation of phase condensates that concentrate the co-activator proteins and the transcriptional apparatus at super-enhancers (153). SRC-3 functions as an AR coregulator in different ways including histone acetylation and RNA Pol II recruitment. The co-operation between SRC-3 and AR is shown to be critical in the regulation of cell cycle-related genes, and its deregulation through overexpression of SRC-3 is shown to be important for PCa cell proliferation, both in the androgen-sensitive and -insensitive stages of the disease (154).

Another protein complex that is important in AR-driven RNA Pol II activation is bromodomain 4 (BRD4). BRD4 is a multifunctional complex with both acetyltransferase and acetylated histone binding functions. It has been shown to both phosphorylate RNA Pol II at the Ser5 position to initiate transcription and also acetylate histones (155). The clinical relevance of AR–BRD4 interplay at enhancers has been shown through the super-enhancer mediated expression of choline phosphotransferase 1 (*CHPT1*), an oncogene whose protein product is an important member of the choline metabolism (156,157). The up-regulation of *CHPT1* is shown to induce resistance to enzalutamide and drive progression to CRPC (157).

AR has also been shown to directly interact with the PIC through TFIIF and affect transcription, potentially through either direct recruitment of TFIIF to the target gene promoter, or by disrupting the TFIIF–RNA Pol II interaction, thereby facilitating promoter escape of RNA Pol II (158,159). In addition to this, the Cdk7 subunit of TFIIH, which carries out Ser5 phosphorylation of RNA Pol II, was identified as an indirect coregulator of AR (160). Cdk7 was shown to phosphorylate the Mediator complex at the Threonine 1457 position of the MED1 subunit and this phosphorylation was shown to stabilize AR–Mediator interaction (160). The kinase activity of Cdk7 was shown to be increased in CRPC, potentially contributing to treatment resistance (160). Cdk7 was also shown to directly phosphorylate AR at Serine 515 to facilitate cyclic transcription of AR target genes by promoting AR turnover through recruitment of MDM2 E3-ubiquitin ligase and directing AR to proteasomal degradation (161).

## Changes to AR that affect its interaction with coregulators

AR gene amplification, emergence of constitutively active variants, and somatic point mutations are all proposed to be drivers of CRPC (162). These changes in AR functionality can dynamically reshape coregulator protein interactions and activity during disease progression. Emergence of ligand-independent variants of AR is a hallmark of CRPC. Of these, AR-V7 is the most well-studied and strongly correlates with drug resistance (163,164). AR-V7 lacks the LBD and is both constitutively active and resistant to current ARPIs (165). Although limited, there are studies comparing the interactomes and cistromes of full-length AR (AR-FL) and AR-V7 (166). Unlike AR-FL, AR-V7 was shown to recruit corepressors such as NCOR1 and NCOR2 to repress tumor-suppressive genes such as *SLC30A7*, *B4GALT1*, *HIF1A*, and *SNX14* (166). In other work, a unique AR-V7 cistrome was shown to be governed by the zinc finger protein ZFX, which is significantly amplified in clinical CRPC samples and plays a critical role in the regulation of specific AR-V7 target genes, cell proliferation, and tumor growth (167). In another study done with exogenously overexpressed protein, it was shown that AR-V7 has a unique cistrome and that variant bound sites have less FOXA1 motif enrichment compared with the sites that are jointly bound by AR-FL and AR-V7 (168). While the AR variant mechanism of action is complex, these findings suggest that AR-FL and AR-V7 have distinct transcriptional coregulators.

AR interacts with FOXA1, HOXB13, and GATA2 through its DBD or NTD (66,83,91). Therefore, it is unlikely that recurrent somatic mutations in the LBD such as L702H, W742C/L, H875Y, and T878A (8) will disrupt these interactions. However, these LBD point mutations alter ligand specificity (169,170). AR activation through non-androgen ligands is potentially the mechanism behind sustained AR activity upon ARPI treatment. However, the impact of these point mutations on AR–coregulator interactions remains to be investigated. In addition, AR overexpression through gene amplification can shift the stoichiometry of AR–coregulator interactions but has not currently been shown to alter AR’s intrinsic affinity to partner proteins.

## Conclusions and future directions

The impact of AR–coregulator interactions on gene expression is an ever-deepening field with each governing mechanism being unveiled like the stacks of a Matryoshka doll. Yet, despite its complexity, our understanding of these regulatory layers continues to expand. This growing knowledge not only refines our current models but also highlights key gaps that remain to be addressed. Answering these questions will deepen our understanding of nuclear receptor transcriptional activity and help to identify novel pharmacological targets against hormone-driven cancers including CRPC. Several major questions that would provide new insight into AR biology include the following:

### Understudied coregulators

Alterations to coregulator interactions change the AR cistrome and initiate oncogenic programs. While well-known coregulators such as FOXA1 and HOXB13 have been extensively characterized, AR interacts with hundreds of other proteins (171). How these understudied coregulators work in concert to drive transcription is poorly understood. We therefore need new high-throughput methods to investigate the functions of AR coregulators. Emerging genomic perturbation techniques that link protein functions to gene regulation pathways, such as PERTURB-seq (172), may be able to provide sufficient throughput to identify the individual roles of coregulators in the initiation and progression of PCa.

### Transcriptional repression

Almost all work has focused on the effects of AR coregulators on gene up-regulation, and the effects of these interactions on AR-mediated down-regulation have been poorly studied. This is likely due to the challenges in the study of transcriptional repression, as well as the controversies of direct AR-repressive activity. However, reports of AR-mediated down-regulation of genes are emerging (173,174). The co-operative action of AR and corepressor proteins is suggested to play a role in the progression of PCa to the hormone refractory state (175–177). With the development of massively parallel reporter assays that can investigate silencing functions, we are now ideally positioned to study the possible impact of AR and coregulators on direct gene repression (178).

### Enhancer–promoter cross-talk

Consistent with their role as enhancers, the majority of ARBS are located in intergenic and intronic regions, and only a very small fraction (1–3%) occur at promoters. It remains unclear how AR coregulators influence target promoters that are not bound by AR. While large protein complexes, like the Mediator complex, can facilitate these long-range interactions, the precise mechanisms remain to be elucidated. A comprehensive understanding is needed of how AR and its coregulators act on RNA Pol II with and without promoter binding.

### Cistrome expansion by AR overexpression

The AR cistrome dramatically changes during CRPC progression and gains >17,000 new sites that reactivate a developmental pathway. A major unresolved question is whether AR overexpression, a key mechanism of ARPI resistance (179), drives this ARBS expansion. It is unclear if this occurs directly through stoichiometric changes to AR motif affinity or by coregulator interactions that affect DNA binding sites.

### Methodological limitations

The genome-wide binding sites of AR and its coregulators are almost exclusively determined by ChIP-seq. However, the limitations of ChIP-seq render the use of complementary approaches necessary to demonstrate a direct link between TF occupancy and transcriptional regulation. Furthermore, AR ChIP-seq is commonly done under super-physiological levels of androgen. While this allows the capture of a large number of binding sites, it does affect context dependency. ChIP-seq data from clinical samples can overcome this issue, but this doesn’t allow for investigation of dynamic AR–chromatin interactions. In addition, the precision in identification of ARBS through ChIP-seq relies on the resolution of the assay, which can vary greatly across experiments due to differences in chromatin sonication. Therefore these approaches only give an overview of larger cis-regulatory elements containing ARBS. Improved resolution of AR-binding sequences in distinct PCa models combined with deep learning approaches can provide new insights into the mechanism of AR binding during disease progression. This requires assays with higher precision, such as ChIP combined with lambda exonuclease digestion followed by sequencing (ChIP-exo) (180) and DNase footprinting assays (181). To determine direct links to gene expression, complementary chromosomal confirmation capture approaches such as Hi-C (182) or HiChIP (183) are required. Further experimental evidence through perturbation of ARBS with CRISPR or CRISPRi can definitively show the impact of AR-target gene regulation by specific CREs. Together, these approaches enable a more precise and functional interpretation of AR-mediated gene transcription.

## Abbreviations

AR: androgen receptor
ARBS: AR-binding sites
ARE: androgen response element
AR-FL: full-length AR
ARPI: AR pathway inhibitor
AR-V7: AR-variant 7
BRD4: bromodomain 4
CHPT1: choline phosphotransferase 1
CRPC: castration-resistant prostate cancer
CTD: C-terminal domain
ChIP-exo: Chromatin immunoprecipitation combined with lambda exonuclease digestion followed by sequencing
ChIP-seq: Chromatin Immunoprecipitation followed by sequencing
DBD: DNA-binding domain
ER: estrogen receptor
GR: glucocorticoid receptor
HAT: histone acetyltransferase
Inr: initiator
LBD: ligand-binding domain
NFI: nuclear factor I
NLS: nuclear localization signal
NTD: N-terminal domain
PCa: prostate cancer
PIC: pre-initiation complex
PTMs: post-translational modifications
RNA Pol II: RNA polymerase II
SELEX: systematic evolution of ligands by exponential enrichment
SRC: steroid receptor co-activator
SRC: steroid receptor co-activator
hARE: half ARE
